# Heterologous Production of Torularhodin, the Monocyclic Carotenoid with a Terminal Carboxyl Group, in *Escherichia coli*

**DOI:** 10.3390/biotech15010003

**Published:** 2026-01-05

**Authors:** Miho Takemura, Takashi Maoka, Norihiko Misawa

**Affiliations:** 1 Research Institute for Bioresources and Biotechnology, Ishikawa Prefectural University, 1-308 Suematsu, Nonoichi 921-8836, Japan; n-misawa@ishikawa-pu.ac.jp; 2 Research Institute for Production Development, 15 Shimogamo-morimoto-cho, Sakyo-ku, Kyoto 606-0805, Japan; maoka@mbox.kyoto-inet.or.jp

**Keywords:** torularhodin, torulene, *Escherichia coli*, *Rhodotorula*, *Planococcus*

## Abstract

Torularhodin is the monocyclic C40 carotenoid with the β-ring and a terminal carboxyl group at the acyclic part, with long conjugated double bonds, only synthesized in fungi called red (oleaginous) yeasts, e.g., the genera *Rhodotorula* and *Sporobolomyces*. This unique red pigment with strong antioxidant properties is promising for use in food additives, nutritional supplements, and cosmetics. We aimed to produce torularhodin in *Escherichia coli* through the identification of the biosynthesis genes needed for its heterologous production, while no genes oxidizing torulene to torularhodin had been reported. The *Rhodotorula toruloides crtI* (*CAR1*) and *crtYB* (*CAR2*) genes, which were chemically synthesized, proved to lead to the complete conversion of phytoene into torulene when they were introduced into an *E. coli* cell that carried the *Pantoea ananatis crtE* and *Haematococcus pluvialis IDI* genes. We found that the *Planococcus maritimus* genes coding for C30 carotenoid terminal oxidase (*crtP*/*crtNb*/*cruO*) and aldehyde dehydrogenase (*aldH*/*crtNc*), through their introduction into the *E. coli* transformant synthesizing torulene, mediated the efficient oxidations of torulene to torularhodin, and resulted in the production of torularhodin as the dominant carotenoid. This is the first report of torularhodin production in a heterologous host. We also identified the *aldH*/*crtNc* gene in *R. toruloides*.

## 1. Introduction

Carotenoids are biosynthesized not only in all photosynthetic organisms that include photosynthetic bacteria, cyanobacteria, algae, and plants but also in a part of non-photosynthetic microorganisms, which comprises bacteria (chemoheterotrophs), archaea, and fungi [1,2,3,4], and even in a few arthropods, e.g., aphids and spider mites [5,6,7]. Their carotenoids are thought to protect their cells from (photo)oxidative stress as a main and common role [8,9]. Among the organisms that can synthesize carotenoids de novo (carotenogenic organisms), only a part of fungi, called red (oleaginous) yeasts, which include the genera *Rhodotorula*, *Rhodosporidium*, *Sporobolomyces*, and *Sporidiobolus*, can produce torularhodin (3′,4′-didehydro-β,ψ-caroten-16′-oic acid) [10]. Among the more than 750 carotenoids that have been isolated from natural sources [1,4], torularhodin is regarded as a unique and special carotenoid due to its monocyclic structure with a typical β ring and an untypical terminal carboxyl group at the acyclic part as well as its long series of 14 conjugated double bonds from the β ring to the carbonyl group. Furthermore, the half-molecule including the β end group is the same as that of β-carotene, indicating that torularhodin can be a vitamin A precursor (provitamin A).

**Figure 1:**
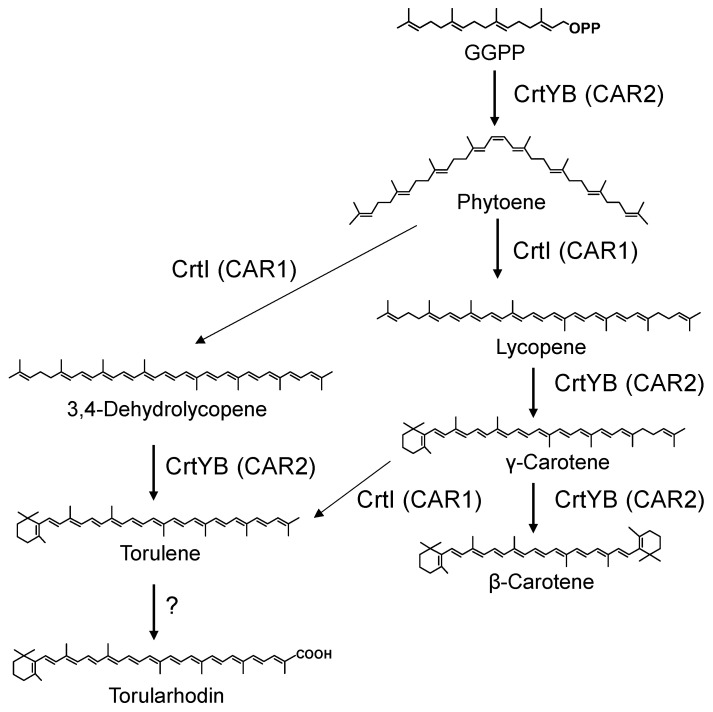
Predicted biosynthetic pathway of torularhodin in the genus *Rhodotorula*. “?” represents the biosynthetic routes that had been unknown. A part of the routes was elucidated in this study, which corresponds to RtAld (see Figure 6 for references).

The unique structural properties of torularhodin among the carotenoids should generate hot research topics among biologists and physiologists. Its strong antioxidant activities have been demonstrated, including activities for scavenging lipid peroxyl radicals and protecting cells from photooxidative damage [11,12,13,14]. The prevention of certain types of cancer has been reported for torularhodin [15]. This pigment is promising for use in food additives, nutritional supplements, or cosmetics [10,15,16]. Current levels of torularhodin in red yeasts (the torularhodin-producing yeasts) are insufficient for commercial production, and genetically engineered red yeast cells as well as knowledge on their carotenoid biosynthesis genes may be necessary to improve torularhodin production levels. The transformation of *Rhodosporidium toruloides* was shown to enhance torularhodin yield [17]. As for biosynthesis, no genes mediating the key steps for torularhodin biosynthesis from torulene have been reported (Figure 1).

The purpose of this study is to produce torularhodin in *E. coli*, hopefully as the dominant pigment. This means that we must identify biosynthesis genes that can be utilized for its heterologous production, while oxidation genes that convert torulene into torularhodin are unknown. We here show the successful production of torularhodin in *E. coli* cells. Through this work, a gene tool for torularhodin production with appropriate hosts should become available for researchers.

All photosynthetic organisms, all carotenogenic non-photosynthetic eukaryotes (fungi and arthropods), and a large part of the other carotenogenic organisms (bacteria and archaea) produce carotenoids that contain isoprene backbones composed of 40 carbon atoms (C40 carotenoids) [1]. On the other hand, a part of the carotenogenic non-photosynthetic bacteria, e.g., all carotenogenic bacteria that belong to the phylum Firmicutes, synthesize acyclic carotenoids with a basic 30-carbon skeleton (C30 carotenoids) [18,19,20,21,22]. Our present study revealed that two genes, which code for C30 carotenoid terminal oxidase and aldehyde dehydrogenase, also mediated the conversion of torulene into torularhodin. The two genes that we used were obtained from a solvent-resistant marine bacterium *Planococcus maritimus* strain iso-3 [22,23]. The C30 carotenoid terminal oxidase and aldehyde dehydrogenase genes have been identified in several bacteria and designated as *crtNb*, *crtP* or *cruO* (described as *crtP*/*crtNb*/*cruO*), and *aldH* or *crtNc* (described as *aldH*/*crtNc*), respectively [20,21,22,23,24,25,26,27].

## 2. Materials and Methods

### 2.1. Microbial Strains

The *E. coli* strain DH5α was used for the cloning. For the expression of the cloned genes, we used the *E. coli* strain JM101 (DE3).

*Rhodotorula toruloides* (JCM10021) was provided by RIKEN BRC (Tsukuba, Japan) through the National BioResource Project of the MEXT/AMED, Japan.

### 2.2. Analysis of Rhodotorula Genome Sequences

A homology search was performed for the *Rhodotorula gracilis* and *R. toruloides* genome sequences using Web BLAST “http://blast.ncbi.nlm.nih.gov/Blast.cgi (accessed on 24 July 2025)”. A motif search was performed using InterPro 107.0 “https://www.ebi.ac.uk/interpro (accessed on 20 October 2025)”.

### 2.3. Construction of Plasmids

Artificial *crtI* and *crtYB* genes of *R. toruloides* (*RtcrtI* and *RtcrtYB*) were chemically synthesized, which were designed to retain modified codons optimized for *E. coli* K12. The *RtcrtI* and *RtcrtYB* genes were amplified by PCR and inserted independently into the *Eco*RI and *Sal*I sites of pUC19. These plasmids were designated as pUC-RtcrtI and pUC-Rt crtYB, respectively. The *RtcrtI* gene was amplified by PCR and inserted into the *Nco*I and *Eco*RI sites of CDF-Duet1. This plasmid was named CDF-RtcrtI. The *RtcrtI* gene was also amplified by PCR and inserted into the *Bgl*II and *Kpn*I sites of the plasmid pAC-HIE, which contained the *Haematococcus pluvialis IDI* (isopentenyl diphosphate isomerase) and *Pantoea ananatis crtE* (GGPP synthase) genes between the *tac* promoter (P*tac*) and the *rrnB* terminator (T*rrnB*) in the pACYC184 vector [28]. The resultant plasmid was named pAC-HIEI. Then, the *RtcrtYB* gene was amplified by PCR and inserted into the *Bgl*II and *Kpn*I sites of the plasmid pAC-HIEI. This plasmid was designated as pAC-HIEIYB.

The *crtP* (*crtP*/*crtNb*/*cruO*) and *aldH* (*aldH*/*crtNc*) genes of the *Planococcus maritimus* strain iso-3 were amplified by PCR using the plasmid pAC-HIMNFNbPA as a template [22,23]. These *crtP* (*PmcrtP*) and *aldH* (*Pmadh*) fragments were independently inserted into the *Nco*I and *Bam*HI sites and into the *Nde*I and *Kpn*I sites of CDF-Duet1, respectively. The constructed plasmids were named CDF-PmcrtP and CDF-Pmadh. Then, the Pmadh fragment was inserted into the *Nde*I and *Kpn*I sites of CDF-PmcrtP. The resultant plasmid was named CDF-PmcrtP-Pmadh. The *aldH* gene of *Rhodotorula toruloides* (*Rtadh*) was chemically synthesized. This gene was amplified by PCR and inserted into the *Nde*I and *Kpn*I sites of both CDF-Duet1 and CDF-PmcrtP. The constructed plasmids were designed as CDF-Rtadh and CDF-PmcrtP-Rtadh, respectively. Using the same restriction sites, we created the plasmid pRSF-PmcrtP-Pmadh. The *CCD* gene of *Rhodotorula toruloides* (*Rtccd*) was chemically synthesized. This gene was amplified by PCR and inserted into the *Eco*RI and *Sal*I sites of pUC19. This plasmid was named pUC-Rtccd.

The structures of these plasmids are shown in Supplementary Figure S1. The accession numbers of the sequences of the plasmids pUC-RtcrtI, pUC-RtcrtYB, pAC-HIEIYB, CDF-PmcrtP, CDF-PmcrtP-Pmadh, pRSF-PmcrtP-Pmadh, CDF-PmcrtP-Rtadh, CDF-RtcrtI, CDF-Pmadh, CDF-Rtadh, and pUC-Rtccd are LC896687, LC896688, LC896682, LC896685, LC896683, LC896686, LC896684, LC902582, LC902583, LC902584, and LC902585, respectively.

### 2.4. Culture of Microbes

The recombinant *E. coli* cells were grown in 10 mL of 2YT medium (16 g/L tryptone, 10 g/L yeast extract, 5 g/L NaCl) containing the appropriate antibiotics such as tetracycline (10 mg/L), spectinomycin (100 mg/L), and kanamycin (40 mg/L) using test tubes at 37 °C with 200 rpm overnight, induced with 0.05 mM IPTG, and further cultured at 20 °C with 200 rpm for 2 days. As for the HPLC/PDA/MS analysis or carotenoid purification, the cells of *E. coli* (JM101) transformants were also cultured in test tubes. A total of 2 L of culture was used for the analysis.

The *R. toruloides* (JCM10021) cells were cultured in 400 mL of YM medium (10 g/L glucose, 5 g/L peptone, 3 g/L yeast extract, 3 g/L malt extract) using 2 L Erlenmeyer flasks.

### 2.5. Carotenoid Extraction and Analysis

Carotenoids were extracted from the recombinant *E. coli* cells with ethanol–chloroform (2:1). HPLC-PDA analysis was carried out as described [29], i.e., the extracted carotenoids were applied to the HPLC-PDA by using a Waters Alliance 2695-2996 system (Waters, Milford, MA, USA) with a column, TSKgel ODS-80Ts (4.6 × 150 mm, 5 μm; Tosoh, Tokyo, Japan). The carotenoid extract was eluted at a flow rate of 1.0 mL/min at 25 °C with solvent A (water–methanol, 5:95, *v*/*v*) for 5 min, followed by a linear gradient from solvent A to solvent B (tetrahydrofuran–methanol, 3:7, *v*/*v*) for 5 min, solvent B alone for 8 min, and then back to solvent A. Max plots (280–560 nm) were monitored. Carotenoids were identified by comparing both their retention times and absorption spectra to those of the authentic standards. All analyses were performed at least twice, and similar results were confirmed.

### 2.6. Purification of Torularhodin

Carotenoid was extracted with acetone from *E. coli* at room temperature and then transferred to diethyl ether (Et_2_O):*n*-hexane (2:8, *v*/*v*) by the addition of water. The Et_2_O:*n*-hexane phase was washed with water several times and was evaporated. The residue was solved with acetonitrile and was subjected to HPLC/PDA/MS analysis [30].

For the NMR analysis, concentrated red residue was dissolved with *n*-hexane and subjected to column chromatography with silica gel 60. The red-colored fraction elucidated with Et_2_O:*n*-hexane (2:8, *v*/*v*) was subjected to preparative HPLC using the Wakosil II 5C18 (4.6 × 150 mm; FUJIFILM, Osaka, Japan) with 0.1% formic acid containing CHCl_3_:CH_3_OH (1:9, *v*/*v*) at a flow rate of 1 mL/min as the mobile phase. The fraction elucidated at 11.2 min was collected. This fraction was evaporated and dried with nitrogen gas. This red residue was then subjected to ^1^H-NMR analysis.

### 2.7. Preparation of Authentic Sample of Torularhodin from Rhodotorula toruloides

The cells of *R. toruloides* (JCM10021) were homogenized with twice the volume of dimethyl sulfoxide for 30 min at 60 °C. Afterwards, carotenoid was extracted with acetone at room temperature. Then, carotenoid was transferred to Et_2_O:*n*-hexane (2:8, *v*/*v*) by the addition of a saturated solution of NaCl in water. The Et_2_O:*n*-hexane phase was washed with water several times and was evaporated. The residue was dissolved with *n*-hexane and subjected to column chromatography with silica gel 60. The red-colored fraction elucidated with Et_2_O:*n*-hexane (2:8, *v*/*v*) was subjected to preparative HPLC using the Wakosil II 5C18 (4.6 × 150 mm) with 0.1% formic acid containing CHCl_3_:CH_3_OH (1:9, *v*/*v*) at a flow rate of 1 mL/min as a mobile phase. Torularhodin was obtained as a fraction eluted at 11.2 min.

### 2.8. Torularhodin Identification

The identification of torularhodin was based on the retention time in HPLC, UV–visible (VIS), negative ion mode electrospray ionization (ESI) MS, and ^1^H-NMR spectral data, in comparison to authentic samples obtained from *R. toruloides*.

HPLC/PDA (photodiode array)/MS analysis was carried out using a Waters Xevo G2S Q TOF mass spectrometer (Waters, Milford, MA, USA) equipped with a Waters Acquity UPLC system. The ESI time-of-flight (TOF) MS spectra were acquired with the negative ion mode. An Acquity 1.7 μm BEH UPLC C18 column (Waters, Milford, MA, USA) was used for the stationary phase of HPLC with a mobile phase of acetonitrile/water (85:15, *v*/*v*)–acetonitrile/methanol (65:35, *v*/*v*) (linear gradient 0–15 min) at a flow rate of 0.4 mL/min. UV-VIS absorption spectra were recorded from 200 to 600 nm using a PDA. The ^1^H NMR (500 MHz) spectrum was measured with a Varian UNITY INOVA 500 spectrometer (Varian, Palo Alto, CA, USA) in CDCl_3_ ushing a Shigemi microtube (sample solution volume 200 mL; SHIGEMI, Tokyo, Japan).

### 2.9. Spectroscopic Data

Torularhodin: UV-vis λmax (Ether) 495, 530 nm; HR-ESI MS; *m*/*z* 563.3911 ([M-H]^−^ C_40_H_51_O_2_, calcd for 563.3889); ^1^H-NMR (CDCl_3_) d 6.14 d (*J* = 16 Hz) H-8, 6.16 overlapped with H-7 and H-10, 6.28 d (*J* = 11 Hz) H-14, 6.31 d (*J* = 11 Hz) H-10′ and H = 14′, 6.36 d (*J* = 15 Hz) H-12, 6.38 d (*J* = 15 Hz, H-6′), 6.34 d (*J* = 15 Hz) H-12′, 6.48 d (*J* = 15 Hz) H-8′, 6.51 dd (*J* = 15, 11 Hz) H-3′, 6.63 dd (*J* = 15, 11 Hz) H-7′, 6.65~6.67 overlapped with H-11, H-11′, H-15, H-15′ and H-4′, 7.38 d (*J* = 11 Hz) H-2′ (Supplementary Figure S2). The data of ^1^H NMR was identical to the reported data [31].

## 3. Results

### 3.1. Torulene Production Using Rhodotorula crtI (CAR1) and crtYB (CAR2)

**Figure 2:**
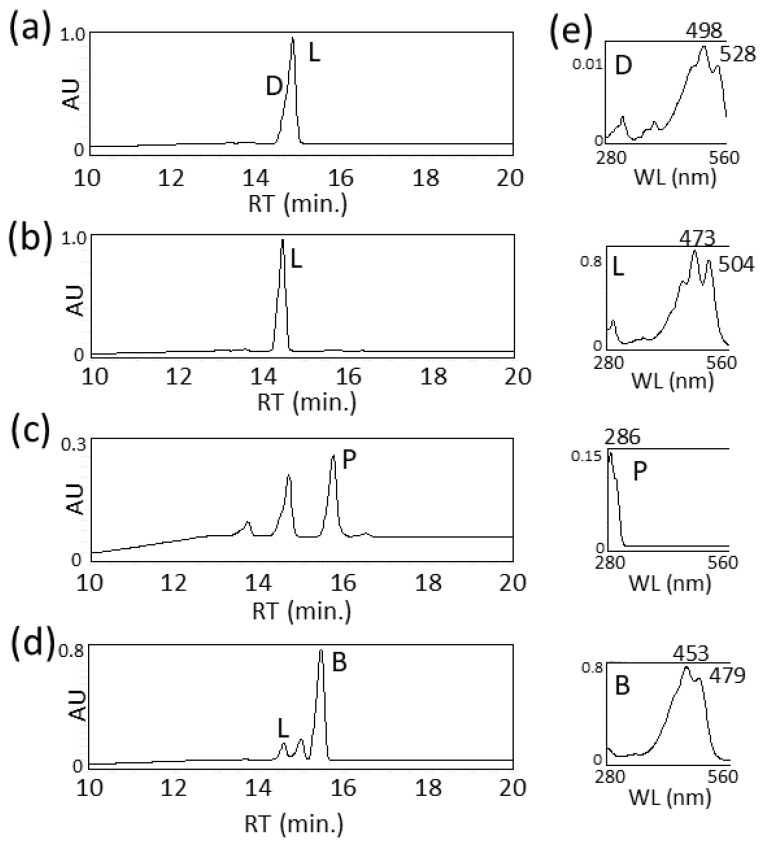
Functional analysis of the *crtI* and *crtYB* of *Rhodotorula toruloides* with *E. coli* cells. HPLC chromatograms of the extracts of *E. coli* transformants carrying pACCRT-EB and pUC-RtcrtI (**a**); pACHP-Lyc and pUC-RtcrtI (**b**); pACHP-GGPP and pUC-RtcrtYB (**c**); and pACHP-Lyc and pUC-RtcrtYB (**d**). (**e**) The UV–visible spectra of compounds, D, L, P, and B. D, 3,4-dehydrolycopene; L, lycopene; P, phytoene; B, β-carotene.

The biosynthetic pathway and genes for torulene have already been elucidated (Figure 1) [16]. Previously, we biosynthesized torulene in *E. coli* using the aphid genes, *ApCrtI4* (*ApTor*) and *ApCrtYB1*, but with low production levels [7]. This time, we noted the *crtI* (*CAR1*) and *crtYB* (*CAR2*) genes of the *Rhodotorula* genus. First, the *Rhodotorula toruloides crtI* (*RtcrtI*) gene (pUC-RtcrtI) was introduced into a phytoene-producing *E. coli* which has the plasmid pACCRT-EB [28], and generated carotenoids were analyzed by HPLC (Figure 2a). As a result, RtCrtI was found to retain activity for synthesizing not only lycopene but also 3,4-dehydrolycopene (3,4-didehydro-ψ,ψ-carotene) from phytoene. To examine whether RgCrtI catalyzes the reaction from lycopene to 3,4-dehydrolycopene, we introduced pUC-RgcrtI into a lycopene-producing *E. coli* which has the plasmid pACHP-Lyc [7]. Carotenoid analysis of this cell extract elucidated that 3,4-dehydrolycopene was rarely produced from lycopene by RtCrtI activity (Figure 2b). This result suggests that RtCrtI produces 3,4-dehydrolycopene from phytoene through consecutive desaturation reactions without accepting lycopene as the substrate. Since the *Pantoea ananatis* CrtI protein, which is included in pACHP-Lyc, shows higher affinity to the CrtB protein-making phytoene, the RtCrtI enzyme is likely unable to synthesize 3,4-dehydrolycopene through the intake of lycopene as the substrate.

Next, we examined the function of the *R*. *toruloides crtYB* (*RtcrtYB*) gene which was predicted to be a dual-functional enzyme. First, the *RtcrtYB* gene (pUC-RtcrtYB) was introduced into a GGPP-producing *E. coli* which has the plasmid pACHP-GGPP [28] and analyzed. The result showed that RtCrtYB catalyzed the reaction from GGPP to phytoene, which is a typical activity of CrtB (Figure 2c). Then, *RtcrtYB* (pUC-RtcrtYB) was also introduced into an *E. coli* which has the plasmid pACHP-Lyc. As a result, RtCrtYB synthesized β-carotene from lycopene, identical to CrtY activity (Figure 2d).

**Figure 3:**
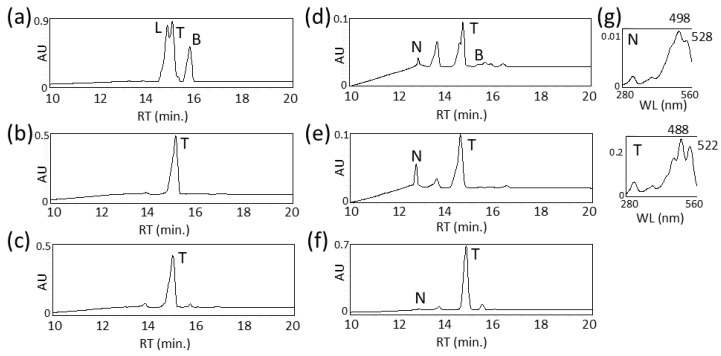
Functional analysis of *crtP* (*crtP*/*crtNb*/*cruO*) and *aldH* (*aldH*/*crtNc*) with *E. coli* cells producing torulene. HPLC chromatograms of the extracts of *E. coli* transformants carrying pACCRT-EB, CDF-RtcrtI, and pUC-RtcrtYB (**a**); pAC-HIEIYB (**b**); pAC-HIEIYB and CDF-PmcrtP (**c**); pAC-HIEIYB and CDF-PmcrtP-Pmadh (**d**); pAC-HIEIYB, CDF-PmcrtP-Pmadh, and pRSF-PmcrtP-Pmadh (**e**); and pAC-HIEIYB and CDF-PmcrtP-Rtadh (**f**). (**g**) The UV–visible spectra of compounds T and N. T, torulene; N, new carotenoid (torularhodin); L, lycopene; B, β-carotene.

Finally, to produce torulene, the plasmids CDF-RtcrtI and pUC-RtcrtYB were co-introduced into a phytoene-producing *E. coli* (pACCRT-EB). As a result, a peak of torulene was detected, but the intermediates lycopene and the biproduct β-carotene were also found (Figure 3a). To increase the production efficiency of torulene, a plasmid containing both the *RtcrtI* and *RtcrtYB* genes with the *IDI* and *crtE* genes (pAC-HIEIYB) was created and introduced into *E. coli*. HPLC analysis revealed that torulene was the predominant product, with a few intermediates such as β-carotene (Figure 3b).

### 3.2. Homology Search in Rhodotorula Genome Sequences

As several *Rhodotorula* genome sequences are publicly available, we first performed a homology search of known carotenogenic genes such as *crtE*, *crtB*, *crtI*, *crtY*, *crtZ,* and so on. Consequently, we found three genes, *crtI* (*CAR1*), *ctrYB* (*CAR2*), and *CCD* (carotenoid cleavage dioxygenase), with significant homology to other corresponding genes. As previously reported, the *Rhodotorula crtI* and *crtYB* genes form a cluster, within which there are *CCD* homolog and unknown genes [32] (Supplementary Figure S3). No other significant homologous genes were found, not only immediately downstream of *crtI* and *crtYB* but also outside this cluster.

### 3.3. Evaluation of Planococcus C30 Carotenogenic Genes

Since we noticed the difficulty of selecting an unknown gene target from the *Rhodotorula* genome sequences, we searched for known genes with similar functions from other carotenogenic microorganisms. Finally, we selected two genes, *crtP* (*crtP*/*crtNb*/*cruO*; here described as *PmcrtP*) and *aldH* (*aldH*/*crtNc*; here described as *Pmadh*), which were isolated from a solvent-resistant marine bacterium *Planococcus maritimus* strain iso-3 producing C30 carotenoids [22,23]. It has been shown that the *crtP* and *aldH* genes code for 4,4′-diaponeurosporene 4-oxidase (4,4′-diaponeurosporene/4,4′-diapolycopene terminal oxidase; aldehyde synthase) [19,24] and 4,4′-diaponeurosporen-4-al 4-dehydrogenase (4,4′-diaponeurosporen-4-al/4,4′-diapolycopen-4-al 4-dehydrogenase; aldehyde dehydrogenase) [20,26], respectively. It was unknown whether these enzymes accept C40 carotenoids as substrates. However, we have noticed that the acyclic half-molecule part of torulene with totally conjugated double bonds completely contains the half-molecular structure of 4,4′-diaponeurosporene or 4,4′-diapolycopene as the CrtP recognition part.

When CDF-PmcrtP was introduced into the torulene-producing *E. coli* (pAC-HIEIYB), no new carotenoid peak was detected (Figure 3c). However, when CDF-PmcrtP-Pmadh was introduced, a new peak was observed around 12.9 min, and its absorption spectra was similar to that reported for torularhodin (Figure 3d). This recombinant *E. coli* contained a significant amount of torulene, and the proportion of torularhodin was low. The peak area ratio of torularhodin, torulene, and β-carotene was approximately 4:95:1. To promote the reaction from torulene to torularhodin, we added the plasmid pRSF-PmcrtP-Pmadh. As a result, the proportion of the new peak increased (Figure 3e); thus, subsequent experiments were conducted using this *E. coli* transformant.

### 3.4. Identification of Torularhodin

**Figure 4:**
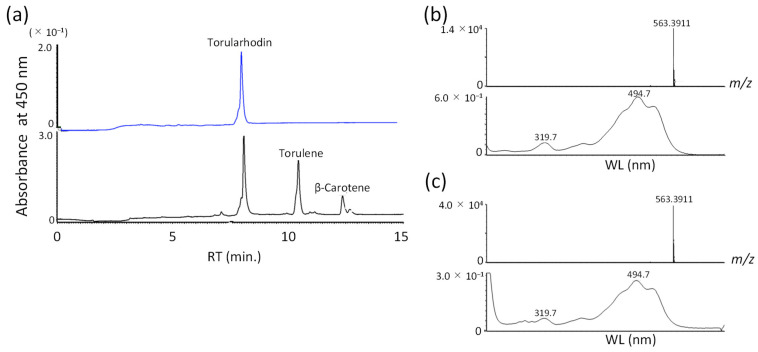
Production of torularhodin in *E. coli*. (**a**) HPLC chromatogram of the extract of *E. coli* transformant carrying pAC-HIEIYB, CDF-PmcrtP-Pmadh and pRSF-PmcrtP-Pmadh (black line) and the HPLC chromatogram of torularhodin purified from *Rhodotorula toruloides* (blue line). (**b**) ESI-MS (upper), UV-VIS spectra (230–600 nm), and maximal absorbance wavelength (lower) of torularhodin purified from *E. coli*. (**c**) ESI-MS (upper), UV-VIS spectra (230–600 nm), and maximal absorbance wavelength (lower) of torularhodin purified from *R. toruloides*.

To confirm the new peak as torularhodin, the red carotenoid was extracted from the recombinant *E. coli* cells, purified, and subjected to the analysis described below. Torularhodin has been difficult to handle due to its free carboxyl group within the molecule and has been identified in forms such as methyl esters [1]. Thus, there are no reports for spectral data of torularhodin as a free form. The identification of torularhodin in *E. coli* was carried out by UV-VIS, negative ion mode ESI MS, and ^1^H-NMR spectral data. VIS absorption maxima showed at 495 and 530 nm (in HPLC mobile phase). The EIS negative ion mode MS showed deprotonated molecules at *m*/*z* 563.3911 [M-H]^−^, compatible with the molecular formula of C_40_H_51_O_2_ (calcd for 563.3889). This MS spectral data directly showed the molecular formula of C_40_H_52_O_2_ for torularhodin. Because of the small amount of the obtained sample and contamination of lipid impurity, the ^1^H-NMR in methyl and methylene signals of torularhodin overlapped with the contamination of lipid signals. Therefore, ^1^H-NMR signals of the olefinic region of torularhodin could be assigned (Supplementary Figure S2). Based on the retention time in HPLC, UV–visible (VIS), negative ion mode electrospray ionization (ESI) MS, and ^1^H-NMR spectral data in comparison with the authentic sample purified from the *R. toruloides* cells, the carotenoid of the new peak was identified to be torularhodin (Figure 4).

### 3.5. Functional Analysis of the aldH Gene from the Rhodotorula Genus

**Figure 5:**
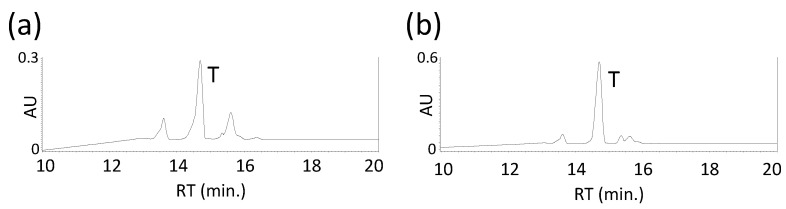
Functional analysis of *adh* genes. HPLC chromatograms of the extracts of *E. coli* transformants carrying pAC-HIEIYB + CDF-Pmadh (**a**); pAC-HIEIYB and CDF-Rtadh (**b**). T, torulene.

We searched again for genes homologous to *PmcrtP* and *Pmadh* in the genome of *Rhodotorula toruloides*. Only *RtcrtI* (*CAR1*) showed significant homology to *PmcrtP*. The failure to produce torularhodin from *E. coli* carrying CDF-Pmadh with pAC-HIEIYB, which includes *RtcrtI* (*CAR1*), suggests that RtCrtI has no terminal oxidation activity (Figure 5a). On the other hand, one gene showing significant homology to *Pmadh* was identified and designated as *Rtadh*. When CDF-PmcrtP-Rtadh was introduced into the *E. coli* producing torulene, torularhodin was produced (Figure 3f). This result demonstrates that RtAdh possesses the same activity as PmAdh to oxidize the terminal aldehyde group of carotenoids. However, since, with RtAdh, it was impossible to produce torularhodin alone without PmCrtP, RtAdh does not possess the same terminal oxidation activity as PmAdh (Figure 5b).

## 4. Discussion

**Figure 6:**
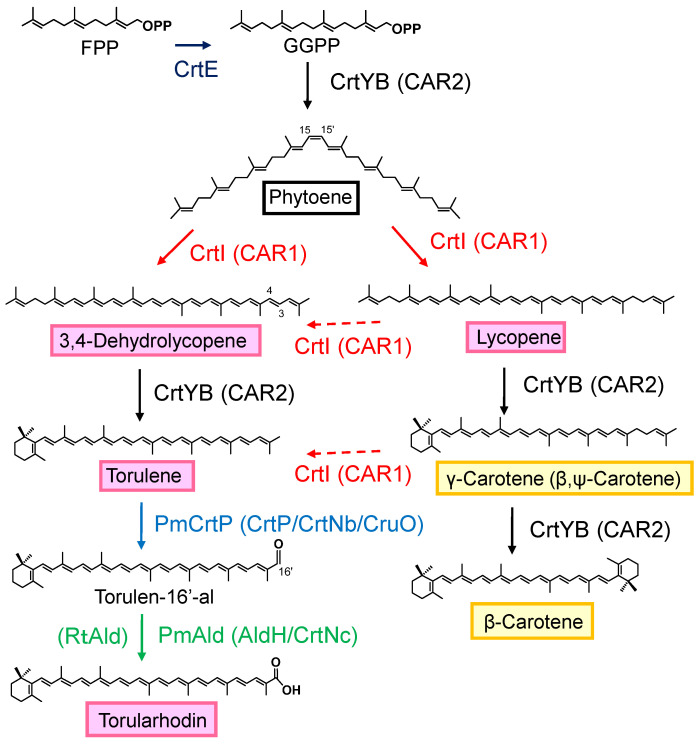
Biosynthetic pathway of torularhodin in the recombinant *E. coli* and functions of the foreign carotenoid biosynthesis genes introduced. Tolulen-16′-al was not observed in this study. CrtI (CAR1) is a phytoene desaturase that catalyzes the consecutive dehydrogenation reactions of phytoene to generate two products, lycopene and 3,4-dehydrolycopene, and is unlikely to accept a generated lycopene molecule as the substrate. CrtI (CAR1) and CrtYB (CAR2) are shown with red and black colors, along with their functions (arrows), respectively. The key routes that come to torularhodin from torulene are mediated by two catalytic functions, terminal oxidase and aldehyde dehydrogenase, which are shown with blue and green colors, respectively.

This study reports the first production of torularhodin in a heterologous host. We produced torularhodin in *E. coli* using the *Planococcus maritinus* genes *PmcrtP* and *Pmadh* (Figure 6). These genes normally act on C30 carotenoids, but our experiment revealed that they are also functional in C40 carotenoids, as long as these possess an acyclic part composed of totally conjugated double bonds. It has also been reported that oxidases such as CrtNb perform the terminal oxidation of C30 carotenoids in *Staphylococcus* and *Methylomonas* [19,20,21,24,25,26]. These enzymes utilize 4,4′-diapolycopene and 4,4′-diaponeurosporene as substrates to produce its aldehydes. Furthermore, the synthesized aldehydes are oxidized into carboxylic acids by aldehyde dehydrogenase (AldH). Tao et al. indicated that *Methylococcus* CrtNb could oxidize the linear ends of the C40 carotenoids, lycopene, and neurosporene with low reactivity [24]. These C40 carotenoids never possess the same acyclic part composed of totally conjugated double bonds as that of C30 carotenoids.

Our previous studies indicated that the *Planococcus maritimus crtP* (*cruO*) gene encodes C30 carotenoid terminal oxidase [23]. At that time, we were able to detect the aldehyde product, 4,4′-diaponeurosporen-4-al (5-hydroxy-5,6-dihydro-diapo-4,4′-lycopen-4′-al). However, in this study, torulene aldehyde (torulen-16′-al; 3′,4′-didehydro-β,ψ-caroten-16′-al) was not detected. Carotenoids with an aldehyde group have rarely been found in nature [1], suggesting that torulene aldehyde should be unstable or harmful to cells including *E. coli*. Alternatively, PmCrtP may exhibit a weaker reaction toward the torulene unless it is together with PmAdh.

The carotenoid terminal aldehyde dehydrogenase gene of *Rhodotorula toruloides* (designated as *Rtadh*), which was selected as a *Pmadh* homolog (ortholog), was also functionally assigned in this study (Figure 6). RtAdh catalyzed the dehydrogenation reaction of torulene aldehyde (torulen-16′-al) into torularhodin, identical to that of PmAdh.

In this study, we were able to identify only three carotenogenic genes, *RtcrtI*, *RtcrtYB,* and *Rtccd*, in the *Rhodotorula* genome. As predicted, RtCrtI and RtCrtYB were involved in the torulene synthesis. CCDs catalyze the cleavage of carotenoids to produce apocarotenoids [33]. We measured the activity of RtCCD; however, as expected, it showed no activity in producing torularhodin (Supplementary Figure S4). The unknown gene in the gene cluster of *Rhodotorula toruloides* was not found in other *Rhodotorula* such as *R. graminis* [21]. In addition, no functional motif was detected in this unknown gene. From these findings, the native terminal oxidase gene was not identified within the *Rhodotorula* genome. This suggests that this terminal oxidase represents a novel gene, presenting an important challenge that requires resolution.

The initial *E. coli* strain engineered for torularhodin production (pAC-HIEIYB + CDF-PmcrtP-Pmadh) resulted in its low productivity (only a few % of total carotenoid) (Figure 3d). When we amplified the expression of the *PmcrtP* and *Pmadh* genes by further adding the plasmid pRSF-PmcrtP-Pmadh, approximately 50% (49%) was torularhodin, 28% was torulene, and 14% was β-carotene among the total carotenoids (Figure 4). The titer of torularhodin was 51 μg/L, whose value has not yet reached higher levels, even compared with that of the red yeasts. This is because their production levels of torularhodin have been increased by improving culture conditions [16,34,35,36].

To further increase the productivity of torularhodin, it would be necessary to adjust tools and methods with *E. coli*, e.g., vectors, promoters, and other elements, as well as to maximize culture conditions. The types of plasmids and promoters are crucial elements that regulate the strength and balance of gene expression. The presence of the intermediate metabolites, torulene and β-carotene, suggests that the expression of the *RtcrtYB*, *RtcrtI*, *PmcrtP*, and *Pmadh* genes is not optimal. Therefore, it is necessary to select plasmids and promoters that achieve optimal gene expression. Optimizing culture conditions is essential for the production of useful substances by *E. coli*. In this study, when 2 L Erlenmeyer flasks were used for culture, torularhodin production dramatically decreased. Therefore, we cultured the bacteria with test tubes. This suggested that better aeration is detrimental to the stability of torularhodin, gene expression, or enzyme function. Therefore, it is necessary to examine the medium components, growth temperature, culture time, and other factors, including aeration.

## 5. Conclusions

This study reports the first production of torularhodin in a heterologous host. The unique monocyclic carotenoid with strong antioxidant properties was dominantly produced in *E. coli* (50% of total carotenoids), which was achieved by using the C30 carotenoid biosynthesis genes *crtP*/*crtNb*/*cruO* and *aldH*/*crtNc* instead of unknown genuine genes. This result should pave the way for the heterologous production of torularhodin with reasonable hosts. This study also identified an *aldH*/*crtNc* ortholog found in the genome of *R. toruloides* as a carotenoid terminal aldehyde dehydrogenase gene, designated as *Rtadh*. On the other hand, we were unable to detect a *crtP*/*crtNb*/*cruO* homolog besides *RtcrtI* (*CAR1*) in the red yeast genome, suggesting that the fungal torulene terminal oxidase gene may retain a structure distinct from the known corresponding enzyme.

## Data Availability

The original contributions presented in this study are included in the article/Supplementary Materials Section. Further inquiries can be directed to the corresponding author.

## Supplementary Materials

The following supporting information can be downloaded at https://www.mdpi.com/article/10.3390/biotech15010003/s1. Figure S1: Schematic diagrams of the plasmids used in this study; Figure S2: ^1^H NMR of torularhodin purified from the recombinant *E. coli*; Figure S3: carotenoid gene cluster of *Rhodotorula toruloides*; Figure S4: functional analysis of the *Rtccd* gene.
