# Supplementary material for: Heterologous Production of Torularhodin, the Monocyclic Carotenoid with a Terminal Carboxyl Group, in Escherichia coli
Source: BioTech (Basel). 2026 Jan 5;15(1):3. doi: 10.3390/biotech15010003 (PMC12821545; doi:10.3390/biotech15010003)
Supplement: Supplementary file 1 [file biotech-15-00003-s001.zip › biotech-4025135-supplementary.pdf]

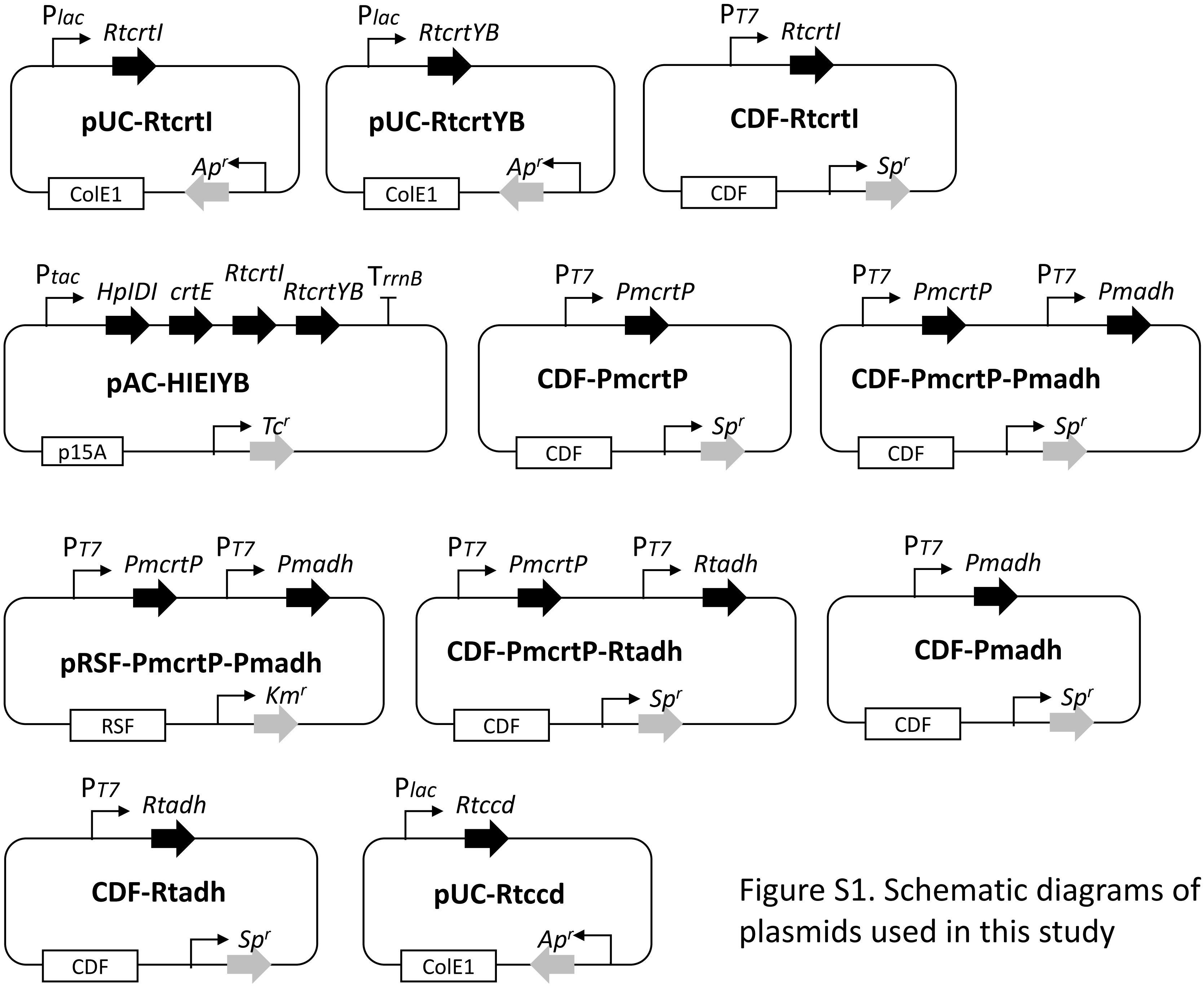

Figure S1. Schematic diagrams of the plasmids used in this study

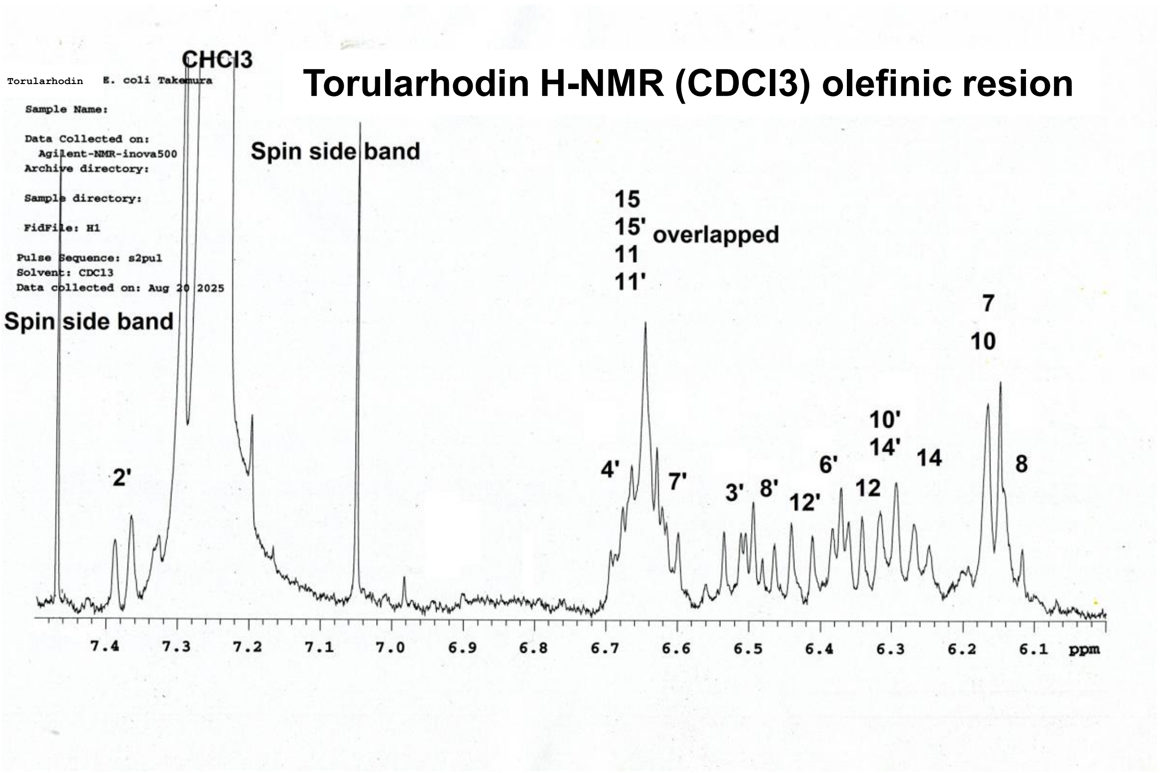

Figure S2. ^{1}H NMR of torularhodin purified from the recombinant *E. coli*

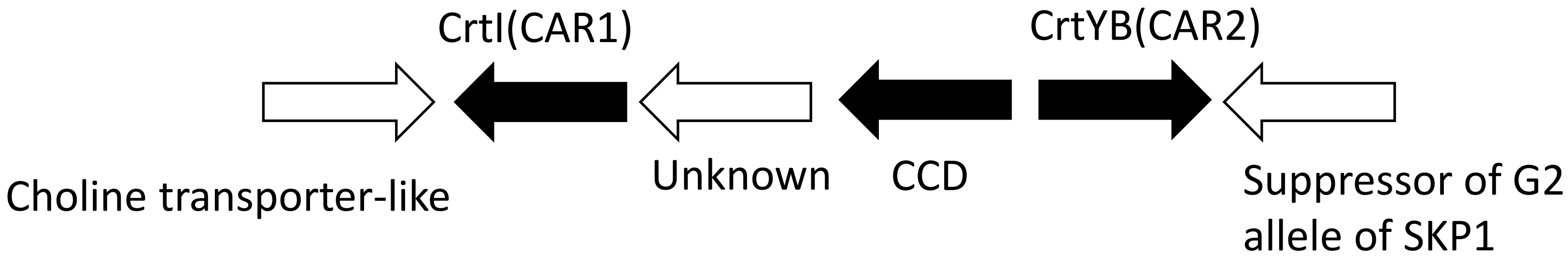

Figure S3. Carotenoid gene cluster of *Rhodotorula toruloides*

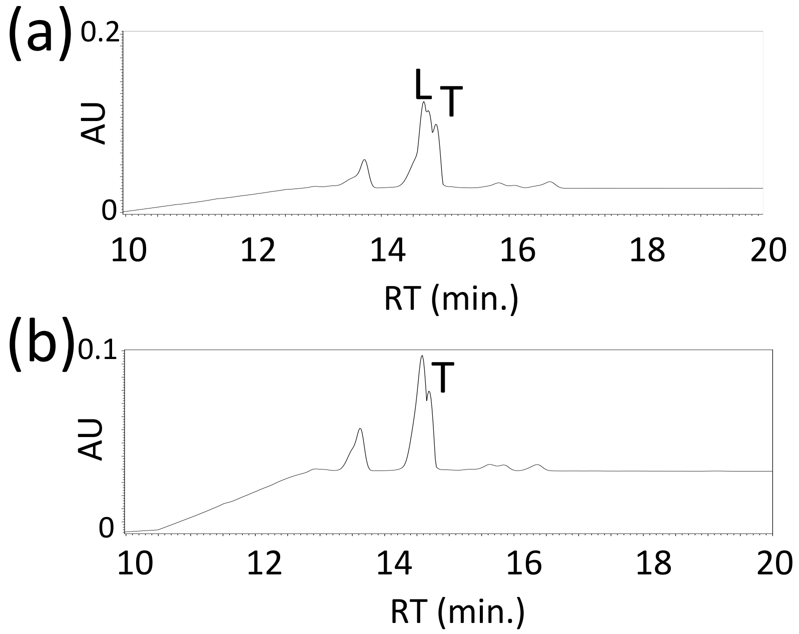

Figure S4. Functional analysis of *RtCCD* gene.
HPLC chromatograms of the extracts of *E. coli* transformants carrying pAC-HIEIYB and pUC-RtCCD (**a**); pAC-HIEIYB, pUC-RtCCD and CDF-Pmadh (**b**). T, torulene; L, lycopene.
